# Can volunteer companions prevent falls among inpatients? A feasibility study using a pre-post comparative design

**DOI:** 10.1186/1471-2318-6-11

**Published:** 2006-08-09

**Authors:** Lynne C Giles, Denise Bolch, Robyn Rouvray, Beth McErlean, Craig H Whitehead, Paddy A Phillips, Maria Crotty

**Affiliations:** 1 Department of Rehabilitation and Aged Care, Flinders University, GPO Box 2100, Adelaide, South Australia, 5001, Australia; 2 Volunteer Service, Repatriation General Hospital, Daws Road, Daw Park, South Australia, 5041, Australia; 3 Nursing Services, Repatriation General Hospital, Daws Road, Daw Park, South Australia, 5041, Australia; 4 Department of Medicine, Flinders University, GPO Box 2100, Adelaide, South Australia, 5001, Australia

## Abstract

**Background:**

Falls in hospital are frequent and their consequences place an increased burden on health services. We evaluated a falls prevention strategy consisting of the introduction of volunteers to 'sit' with patients identified as being at high risk of falling.

**Methods:**

Two four bed 'safety bays' located on medical wards in two hospitals within southern Adelaide were used. Ward fall rates (expressed as falls per 1000 occupied bed days) were compared in the baseline period (February-May 2002) with the implementation period (February – May 2003) using incident rate ratios and 95% confidence intervals. The number of hours of volunteered time was also collected.

**Results:**

No patient falls occurred on either site when volunteers were present. However, there was no significant impact on overall ward fall rates. In the baseline period, there were 70 falls in 4828 OBDs (14.5 falls per 1000 OBDs). During the implementation period, there were 82 falls in 5300 OBDs (15.5 falls per 1000 OBD). The IRR for falls in the implementation versus baseline period was 1.07 (95%CI 0.77 – 1.49; P = 0.346). Volunteers carried out care activities (e.g. cutting up food), provided company, and on occasions advocated on behalf of the patients. Volunteers donated 2345 hours, at an estimated value to the hospitals of almost $57,000.

**Conclusion:**

Volunteers may play an important and cost-effective role in enhancing health care and can prevent falls in older hospital patients when they are present. Full implementation of this program would require the recruitment of adequate numbers of volunteers willing to sit with all patients considered at risk of falling in hospital. The challenge for future work in this area remains the sustainability of falls prevention strategies.

## Background

Falls in hospital and their consequences place an increased burden on health services [1-3]. While falls are frequent, accounting for 38% of incident reports in Australian hospitals [4], relatively few studies have demonstrated effective strategies to reduce falls among inpatients [5]. A systematic review of the falls prevention literature showed that while there are a number of interventions likely to be beneficial, more research is required [6].

Commonly identified falls risk factors in the hospital setting include impaired cognition, impaired mobility, prior falls history, medications, incontinence, and advanced age [7]. Falls occur at any time of the day, although they often coincide with peak periods of activity in the ward such as mealtimes [3]. It has been reported that as many as 68% of recorded falls in hospital are not witnessed [3].

While interventions targeted at the individual level are appropriate to prevent falls in the community, the frequency of inpatient falls justifies interventions that assume all patients are at risk and are aimed at the total ward population. Two major approaches at this level are either to redesign the physical environment of the ward or provide more staff, especially at times known to be high risk for falling. Both of these approaches require additional funding. An alternative strategy to reduce falls among inpatients is increased patient observation, particularly in areas such as the patient's bedside and room area, bathroom, toilet and corridors, all of which have been shown to be common locations of falls [1,8,9].

The design of most hospital wards and nursing staffing levels limits the ability of nurses to provide intensive surveillance of patients at risk of falling [10]. Another option is to recruit hospital volunteers to sit with patients identified as at high risk of falling. Volunteer patient companions have been used in the palliative care setting [11] and are recommended in current Australian best practice guidelines for falls prevention in hospitals [12], but there is a paucity of literature concerning the effects of volunteer companions on falls rates in the acute care setting. No randomized controlled trial has examined the effects of volunteer companions on falls rates in hospitals. While such a trial would provide the optimal evidence, feasibility studies of volunteers acting in this way are first needed.

In a recently reported study conducted in New South Wales [10], patients considered at high risk of falling were place in a four bedded room near the nurses' station. Volunteers worked from 8 am to 8 pm on weekdays. Volunteers worked in pairs, with one volunteer canvassing the ward for wandering patients, and the other remaining in the observation room. The authors concluded that approximately six falls were prevented every month by the volunteers.

In 2002, we began a project utilising volunteers to reduce falls. In the acute care setting, we envisaged that the volunteers would provide diversional and reminiscence therapy and summon help for the patient if needed. This would allow for additional observation of the patients and reduce the frequency of patients leaving their beds whilst unattended, therefore decreasing the patients' opportunities to experience a fall.

The primary aim of the project was to examine the effectiveness of the increased patient observation, via a program of volunteer patient companions, in reducing falls among inpatients. A secondary aim was to evaluate the acceptability of the volunteer patient companions program from the perspective of the volunteers, the patients' families and hospital staff.

## Methods

### Program development

The volunteer companion initiative started in July 2002 in two public hospitals in Southern Adelaide, totaling 370 beds. The hospitals were two of the three public hospitals in the southern region of Adelaide involved in the National Demonstration Hospital Program, Phase 4 (NDHP-4) conducted in 2002–3. The wards considered in the present study were the major geriatric wards in the two hospitals. A project team that included medical, nursing, and allied health staff, two consumer representatives, project representatives, and volunteer coordinators from across the two sites was established to develop a description and clear guidelines for the volunteer companion role (see Table 1). Consultations were held with key stakeholders including clinical staff, hospital management and appropriate union bodies, and issues such as occupational health and safety for volunteers and the potential for the volunteer companions' role to impinge on duties undertaken by paid staff were discussed.

**Table 1:** Description of the Volunteer Companion Role

*Role description*	• The volunteer companion is an unpaid member of staff, whose primary function is to observe those patients at high risk of falling.
*Purpose of Role*	• Observe patients at high risk of falling and notify nursing staff of all potential occasions where the patients may fall. • Engage patient in social interaction and perform diversional activities as appropriate. • Complement the roles of paid staff thereby enhancing service provision.
*Activities*	• Observe patients in the allocated patient bay and intervene as required to minimise the risk of patients falling. • Recognise and report immediately any change in patients' behaviour, which could increase the risk of harming themselves or others. • Social interaction with patients is encouraged. • Diversional activities including reading to patients, reminiscence, singing. • Recreational activities including taking patients for a walk in the wheelchair. • Helping activities including pouring a drink, cutting up food. • Therapeutic activities e.g. hand massage.
*Activities not to be performed*	• 'Catching' a patient who is falling. • Patient feeding. • Patient transfer (from bed to chair/wheelchair). • Patient toiletting.
*Responsibilities*	• Undertake the volunteer companion orientation and training program. • Volunteer for one four-hour shift each week for the duration of the trial. • Ensure that all patient information is treated confidentially. • Respect the values, customer and spiritual beliefs of patients. • Demonstrate sensitivity to privacy and dignity aspects of patient care. • The volunteer is covered by the hospital's workers compensation policy.
*Reporting*	• The volunteers report to the hospital volunteer coordinator but works under the direct supervision of the Registered Nurse. • Nursing staff communicate relevant patient information at the commencement of the shift.
*Remuneration*	• Nil, including no payment of expenses (e.g. Transport)

### Implementation

A pre-post comparative design was used to determine whether the introduction of a 'safety bay' for patients identified at high falls risk impacted on ward fall rates. At each site patients considered to be at highest risk of falling were allocated to a four bed 'safety bay' where the volunteer companions could closely observe them. At one site, the safety bay was located in a general medical ward, whereas the safety bay was located within a ward that accommodated patients with dementia and behavioural problems at the second site. To identify high-risk patients, one site used the STRATIFY risk-screening tool [13] while the second hospital relied on clinical judgement. This was the standard protocol in each of the hospitals.

The volunteers observed patients in the safety bay from 9 am to 5 pm, Monday to Friday at both sites, with a four hour shift on Saturday mornings also provided at one of the sites. Each volunteer worked a four-hour shift either in the morning or afternoon, and most volunteered one shift per week. In the general medical ward setting, the safety bay was attended by one volunteer at a time, whereas at the second hospital two volunteers at a time worked together. Working in pairs was at the request of the volunteers as the patients with dementia and behavioural problems needed a much higher level of observation. Volunteer companions reported to the volunteer co-ordinator on each site.

### Recruitment and training of volunteers

Volunteers were recruited through advertising in the local newspaper and through city councils. Information sessions for prospective volunteers were held to provide details about the project. A compulsory three-day training program was developed and included general hospital orientation, issues regarding confidentiality, information on falls prevention, and sessions to prepare the volunteers for working with older, confused patients who often suffered with dementia.

### Monitoring and evaluation

Details of falls were recorded in the hospital's Advanced Incident Monitoring System (AIMS). The use of AIMS in South Australian public hospitals is mandated by the South Australian Department of Health. The rate of falls per occupied bed day (OBD) for the February – May 2002 (baseline period) and February – May 2003 (implementation period) at each site was compared using incidence rate ratios (IRRs) and 95% confidence intervals (CI). The volunteer companion initiative began in July 2002 and the remainder of the year was spent in recruiting and training volunteers at the two hospitals. Volunteers were formally implemented in the wards in February 2003. The implementation period was constrained to four months because the final report to the Commonwealth Government regarding the NDHP-4 projects were required in August 2003. The time of day (classified as 7:00 am-10:59 am, 11:00 am-2:59 pm, 3:00 pm-6:59 pm, 7:00 pm-10:59 pm, 11:00 pm-2:59 am, 3:00 am-6:59 am) and day of the week of falls pre- and post- introduction of the volunteer companions were compared using chi-square tests of association. The demographic profile of fallers before and after the introduction of volunteer companions was compared using chi-square tests of association and Mann-Whitney U tests.

Qualitative data were collected to evaluate the experience of the volunteer program for the volunteers, patient's families and hospital staff. Data sources included journals that the volunteers kept to document their experiences throughout the project, volunteer satisfaction surveys, semi-structured telephone interviews with patients' families, and staff satisfaction surveys.

Volunteers' journals were transcribed, and content analysis was then undertaken by two independent researchers to identify major themes. The satisfaction surveys included open-ended questions about the volunteer role, and satisfaction with the role from the perspective of both the volunteers and the ward staff. The responses to the satisfaction surveys were summarised using counts and percentages. Telephone interviews with members of patient's families were conducted by a research nurse. They were not tape recorded, as it was thought this may inhibit the interaction between the interviewer and the family. Notes were taken during the telephone interviews. The interviewer validated each response by reading the notes taken back to the family member, who was able to clarify and add to their response before moving on to the next question. Content analysis of the notes was used to identify recurrent themes in the family interviews, and was undertaken by the same researchers that considered the volunteers' journals.

Hours of time, including time spent in training, donated by volunteers were estimated retrospectively from rosters held by the volunteer coordinator at each site. The estimated value of donated time was calculated using the casual hourly rate of $AU24.25 for an enrolled nurse in the South Australian Government Public Sector. This rate of pay included an allowance of 20% for superannuation and other loadings. Estimates of travel time and transport costs were not included.

All patients (or their proxy), family members, staff, and volunteers who participated in this study gave written informed consent. Ethical approval for this study was obtained from the Repatriation General Hospital Research and Ethics Committee and the Noarlunga Health Service Research and Ethics Advisory Group.

## Results

A total of 45 volunteers were recruited for this project, with the majority of volunteers aged more than 65 years, and more females volunteering than males. Thirteen volunteers left the project before the implementation period was completed, with the major reasons cited as dissatisfaction with the role (n = 5), opportunity for paid work or study (n = 4), and only a short-term time commitment made or other reason (n = 4).

### Falls rates

**Figure 1:**
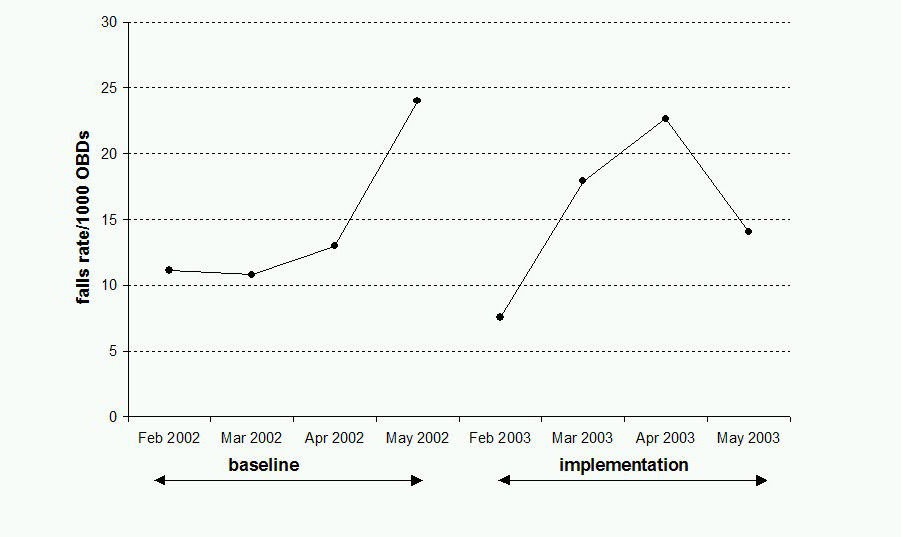
Falls rate per 1000 Occupied Bed Days in baseline and implementation periods.

Figure 1 displays the falls rate per 1000 OBDs for each month in the baseline and implementation periods. In the baseline period, there were 70 falls in 4828 OBDs (14.5 falls per 1000 OBD). During the implementation period, there were 82 falls in 5300 OBDs (15.5 falls per 1000 OBD). The IRR for falls in the implementation versus baseline period was 1.07 (95%CI 0.77 – 1.49; P = 0.346). No falls occurred in the wards at either site when the volunteers were present. However, 24% of falls in the wards during the implementation period occurred in the safety bays when volunteers were not present, indicating the safety bay alone was not sufficient to prevent falls.

There was no significant difference in the distribution of falls over days between the baseline and implementation periods (χ^2 ^= 8.8, 6 df; P = 0.18). In addition, there was no significant difference in the distribution of falls over time between the two periods (χ^2 ^= 9.1, 5 df; P = 0.11). The profile of fallers in the two years was not significantly different in terms of age (P = 0.482), gender (P = 0.513) or length of hospital stay (P = 0.912).

### Volunteers' perspectives

Nineteen volunteers (60% of volunteers retained) completed journals. A total of 33 journal entries commented on the volunteer role in regard to falls prevention, and all comments were positive about the volunteer companion's role:

"*I went over and encouraged him to stay on his bed so that he would not topple over*."

"*I had to ring the emergency bell 8 times, with clients getting out of bed so we did prevent some patients falling today*."

"*Patient tried to go to the toilet by himself, I called for a nurse and persuaded him to wait. This I think was a positive fall save*."

The journals also showed that volunteers enjoyed their role:

"*Each Tuesday morning I look forward to this outing. Meeting different people each week and one hopes to contribute something to make their stay in hospital pleasant and NO FALLS*."

"*The volunteers' presence seems to have a calming effect on the behaviour of the patients and also that the nursing staff have requested that more volunteers be recruited. A very nice result for our efforts!*"

Sixteen volunteers (50%) responded to a survey at the end of the implementation period concerning their satisfaction with their role. All respondents reported they were satisfied with the role as a volunteer companion, all agreed that their role had been useful in preventing falls, and all respondents agreed that the volunteer companion role should continue.

### Family interviews

All families that were approached agreed to the semi-structured telephone interview (10 families from each site). There was generally poor understanding about the purpose of the volunteer companions, with only eight of the families aware that the volunteers' role was to prevent falls. Four respondents thought the volunteers were to "*help with various housekeeping chores*" or "*to provide support to the patients and help them*". Eight respondents did not know why the volunteers were present.

Although there was confusion about the purpose of the volunteer role, no negative comments were made concerning the volunteers. All 20 family members were positive about the volunteers and reported the volunteers provided company and help to the patients, as the following quotes illustrate: "*We thought they were brilliant, my dad has such poor eyesight and limited mobility and the volunteers were really helpful*."

"*Wonderful people, helped with the small things, showed me where to go and where the kitchen was*."

"*Wonderful company for the patients, even the dementia patients have some company so they are not lonely*."

### Nurse satisfaction

All nurses that worked with the volunteers (n = 24) completed a survey about the volunteer role at the conclusion of the implementation period. All respondents stated that they understood the volunteer role, and 22 (92%) respondents agreed that the introduction of the volunteer companions was useful as a falls prevention strategy, and thought that the role should continue. However, 7 (29%) respondents claimed that the volunteer companion role required too much supervision. One comment illustrated this perception:

"*Sometimes quite demanding of staff time, interrupt inappropriately for minor problems eg. during drug rounds...*"

#### Donated value of volunteers' time

Volunteer companions at the two hospitals donated 2345 hours during the implementation period. Costed at $AU24.25 per hour, this represents a total value of $56,866. This figure does not include estimates of travel time per volunteer nor transport costs.

## Discussion

These results indicate that the implementation of a volunteer companion program can prevent falls *when the companions are present*. The project aimed to reduce the incidence of falls by the introduction of observers and resulted in no falls reported in the safety bay in the hours that the volunteers worked. However this did not reduce the incidence of falls outside of the safety bay during the hours that the volunteers worked or after hours both in the safety bay and in the remainder of the ward.

Similarly, the comparable NSW trial [10] demonstrated no falls occurred in the observation room when volunteers were present. These authors also demonstrated a 44% reduction in falls risk on the ward overall, but the falls rate escalated when volunteers were not present in December and January. Furthermore, volunteers provided an extra 20 hours of observation per week in this study, and a 'roving' volunteer assisted inpatients who wandered. These differences from our study may explain why we were unable to demonstrate a reduction in falls on the ward overall.

Taken together, the studies suggest a parallel may be drawn with strategies designed to reduce falls sequelae, such as hip protectors. Hip protectors may be effective in preventing hip fractures but only while being worn [14]. Volunteer companions may thus be viewed as a useful addition to a set of multiple falls prevention strategies [15].

While the primary focus of the project was to reduce falls it was acknowledged in project planning that the volunteer companion role would include other 'value-added' activities for patients to occupy their time. The impact of these other role dimensions on the patients, patients' families, nursing staff and volunteers themselves was underestimated. Companions acted as patient advocates, provided companionship, and enhanced the delivery of patient care. Although many families or carers were unaware that the volunteers were in the safety bay to prevent falls, they all commented on the contribution the volunteers made in providing company for their relative. In particular, the families commented that they could leave, knowing that their family member would have company in their absence. However, it is important that any implementation of volunteer companions in falls prevention or other settings makes their role very clear to patients and their families.

Not only was the quality of care improved for patients during the implementation period, but the volunteers were very satisfied with their participation in the program. This finding is supported by a meta-analysis on volunteering which suggests that volunteers enjoy a greater quality of life than non-volunteers [16]. An Australian review that examined the relationship between volunteering and health among older people suggests that these positive benefits extend to health outcomes, including reductions in morbidity and improvements in self-reported health [17]. This enhanced quality of life for volunteers could be used as recruitment strategy in the future.

An important factor in sustainability of the volunteer companion program was the incorporation of the program within existing hospital structures and processes, resulting in minimal additional costs. The volunteer coordinators on both sites were integral project team members and ensured that the companion program management could be incorporated within the existing volunteer service at each hospital.

There were a number of limitations in the research reported here. A randomized controlled trial would have provided the best evidence concerning the effects of volunteer companions on falls rates. However, it would be premature to embark on such a trial before the feasibility of volunteer companions was established, and a comparative design was the most pragmatic option to assess their feasibility. It is also possible that staff were reluctant to report some falls during the implementation period. However, the reporting of falls is mandated by the State Department of Health, and the falls rate was slightly higher during the implementation period than in the baseline period. It is thus unlikely that an ascertainment bias in the reporting of falls occurred.

The major limitation to sustainability remains the capacity to recruit and retain adequate numbers of volunteers. Seven days a week, twenty-four hour a day monitoring of the safety bay would require an additional fifteen volunteers per ward. Expanding this concept to observe other at risk patients would see this figure rise significantly. Attempts to recruit volunteers to sit with patients overnight are unlikely to be successful. The recruitment campaigns undertaken during the study demonstrated clearly that this number of volunteer companions would be unachievable without re-examining our volunteer profile or safety bay concept. The potential to expand the recruitment pool may be possible through attracting an alternative volunteer profile such as students or unemployed people, as was successful in the NSW study [10]. However research indicates that older people are more likely to volunteer because it provides them with social interaction [18,19]. The attrition rates of volunteers differed at the two hospitals in our study, and the main reason for this appeared to be in the different approaches to education about the volunteer role at the outset of the program. Along with effective recruitment strategies, volunteer selection, training and on-going education are essential to the continued success of initiatives such as this. Remuneration of transport costs may also assist in the recruitment and retention of volunteers.

The strategy was also limited by confusion about the role of the volunteers among patients' families. Dissatisfaction among nursing staff about the level of supervision the volunteers required highlighted that any information about the volunteer companions that is disseminated to the hospital community must be carefully developed. Furthermore, the current design of most traditional ward settings limits the observation of patients that is achievable unless direct patient care activities are in progress.

## Conclusion

Our project demonstrates that while preventing falls in hospital is difficult and requires significant and sustained culture changes in the practice setting, volunteer companions can play a role in enhancing the care of older Australians during their hospital stay. Such programs should be actively encouraged and supported with creative solutions developed for expanding the pool of these valued community members.

## Competing interests

The author(s) declare that they have no competing interests.

## Authors' contributions

LCG participated in the design of the study, performed the statistical analyses, and drafted the manuscript. DB assisted in the study design and coordination, data collection and drafted the manuscript. RR managed the volunteer service and assisted in data collection. BM assisted in data collection and extraction of falls data from the hospital critical incidents' system. CHW participated in study design and drafted the manuscript. PAP participated in study design and drafted the manuscript. MC conceived of the study, and participated in its design and coordination and drafted the manuscript. All authors read and approved the final manuscript.

## Pre-publication history

The pre-publication history for this paper can be accessed here:


